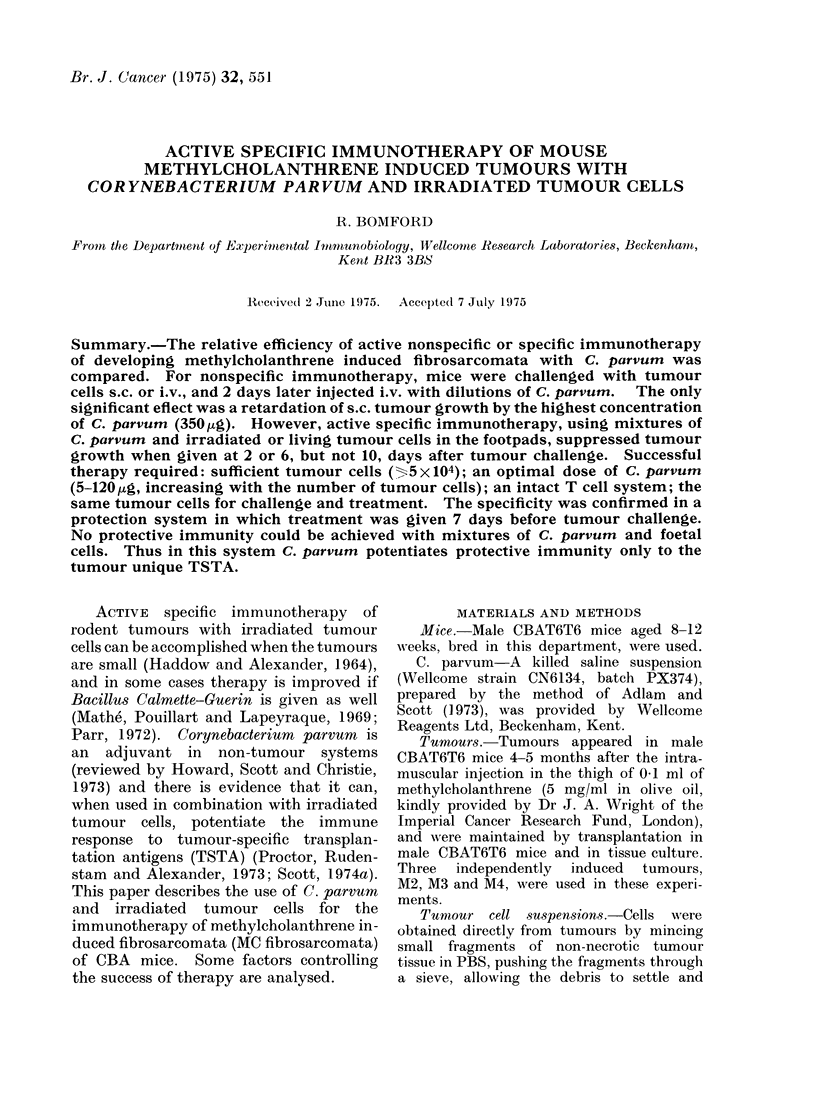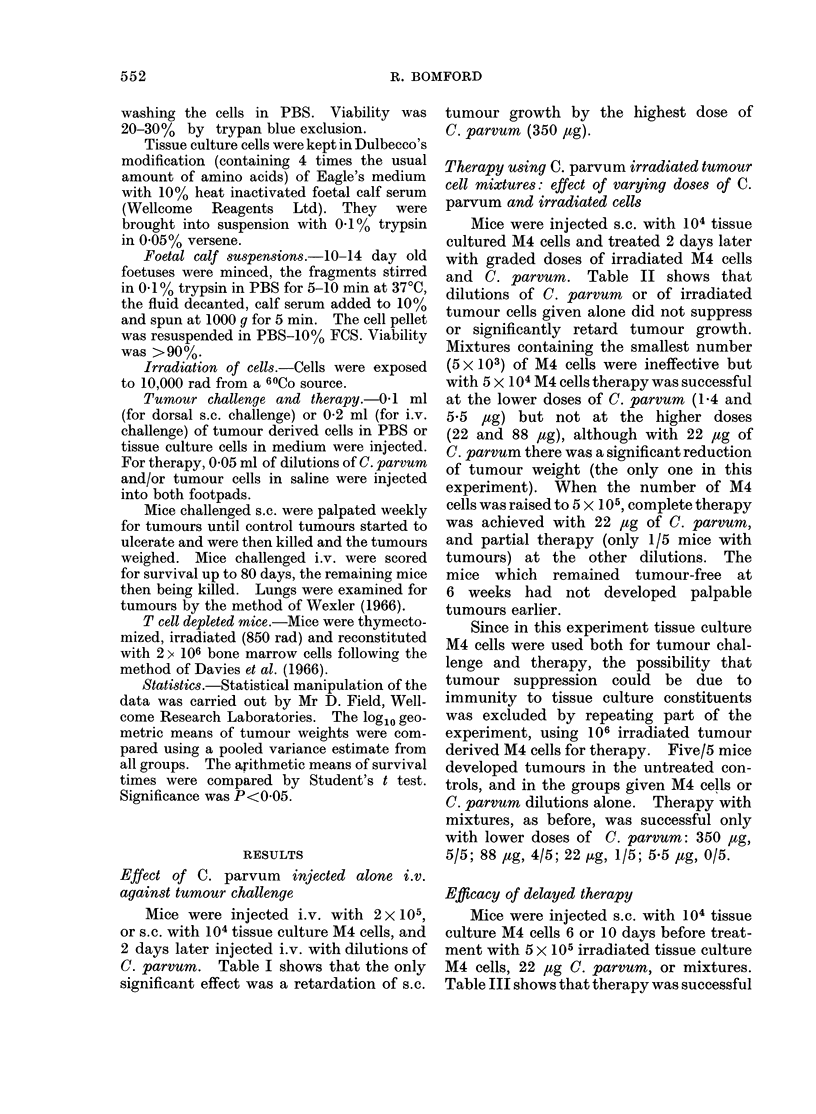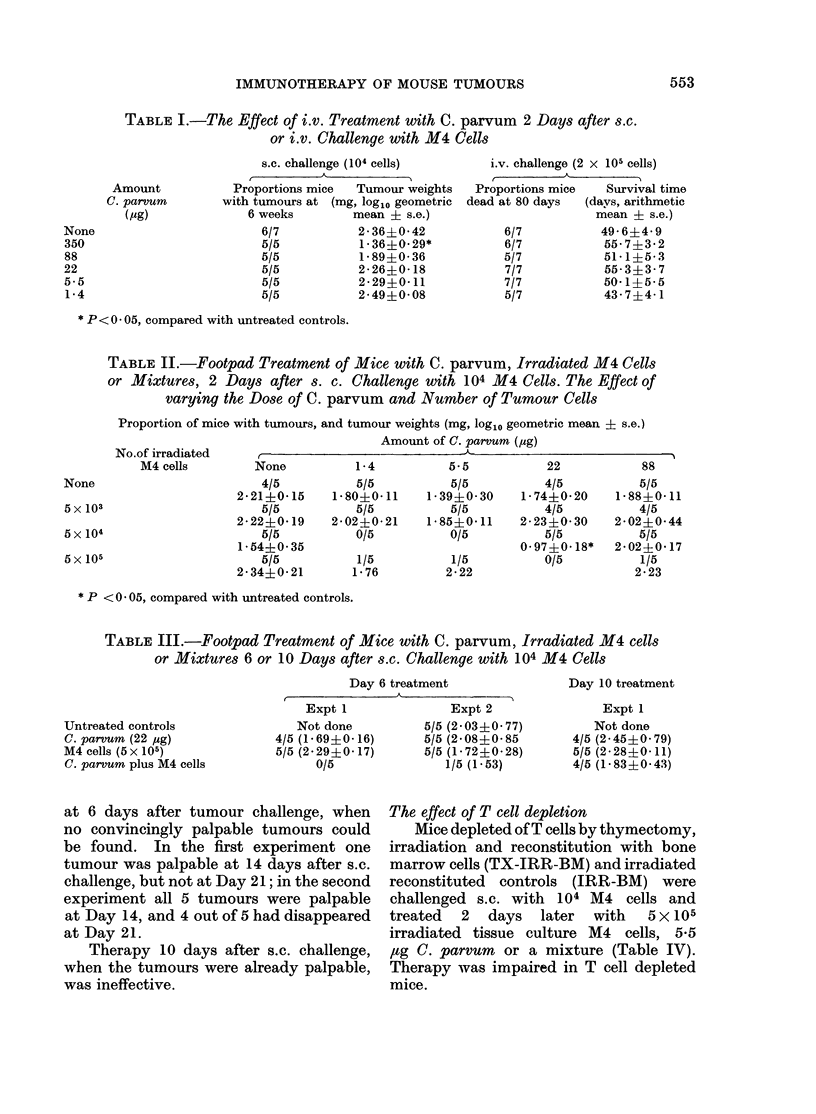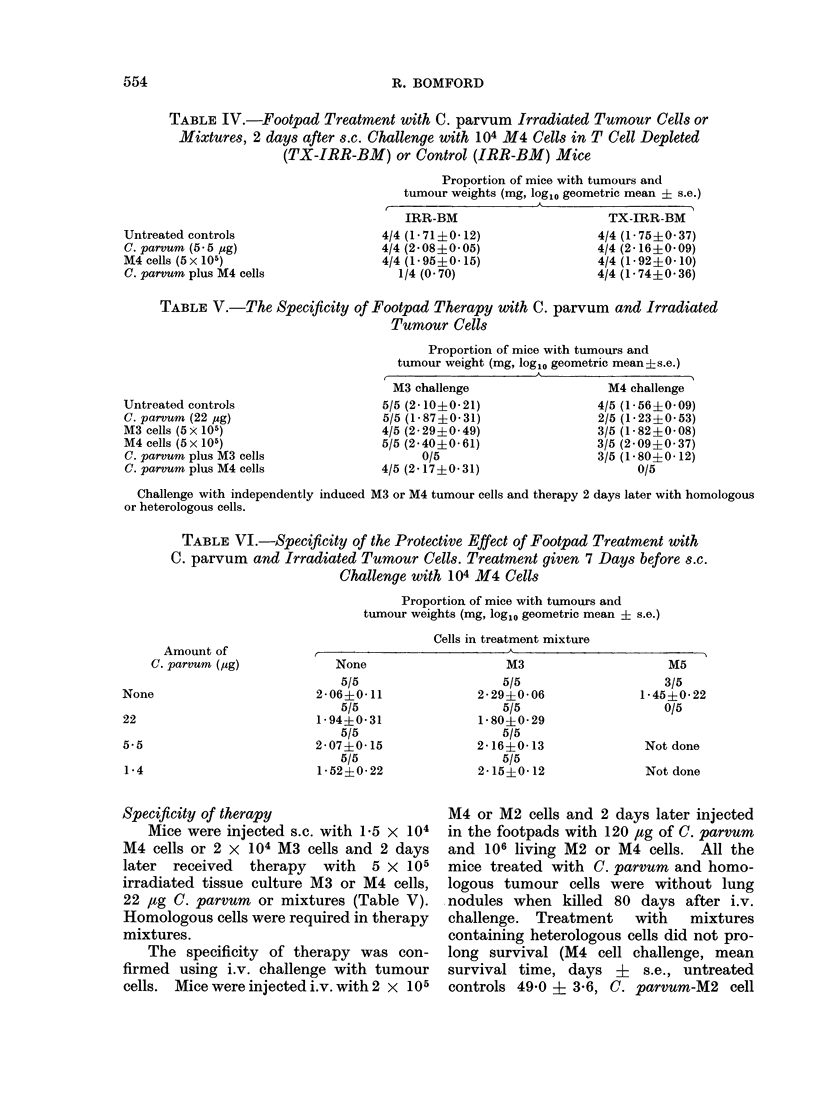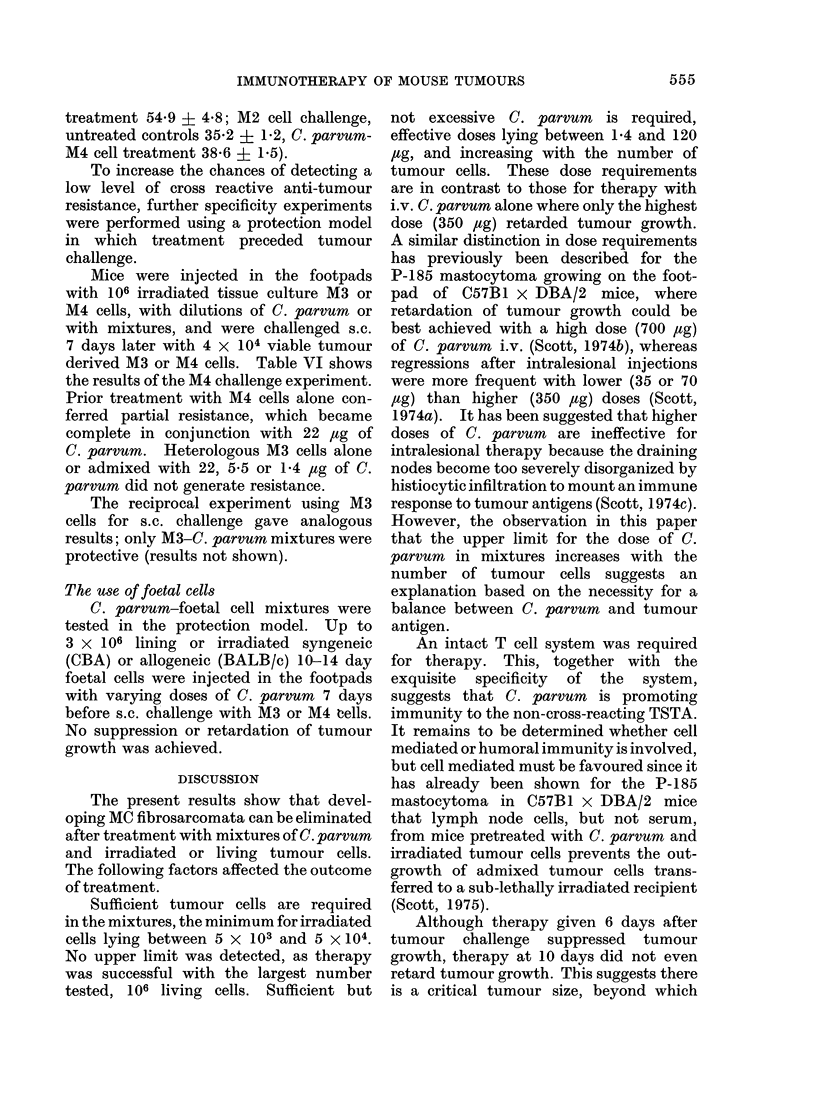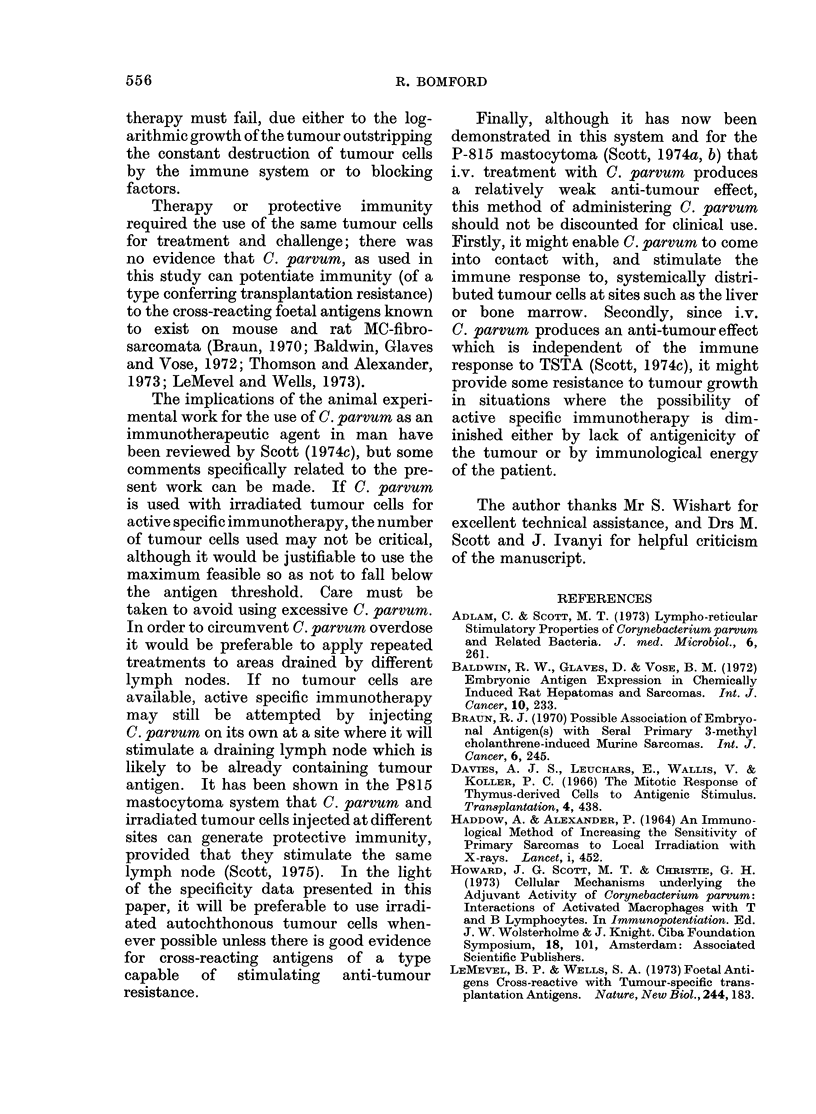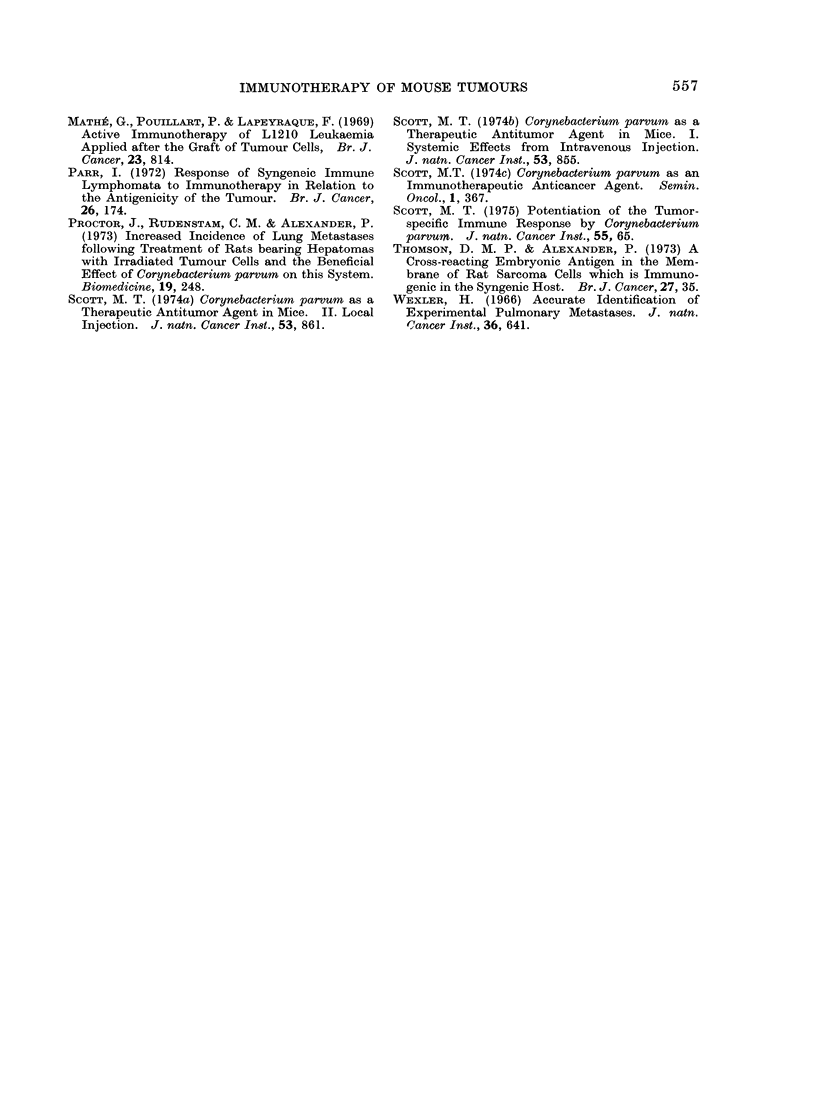# Active specific immunotherapy of mouse methylcholanthrene induced tumours with Corynebacterium parvum and irradiated tumour cells.

**DOI:** 10.1038/bjc.1975.260

**Published:** 1975-11

**Authors:** R. Bomford

## Abstract

The relative efficiency of active nonspecific or specific immunotherapy of developing methylcholanthrene induced fibrosarcomata with C. parvum was compared. For nonspecific immunotherapy, mice were challenged with tumour cells s.c. or i.v., and 2 days later injected i.v. with dilutions of C. parvum. The only significant effect was a retardation of s.c. tumour growth by the highest concentration of C. parvum (350 mug). However, active specific immunotherapy, using mixtures of C. parvum and irradiated or living tumour cells in the footpads, suppressed tumour growth when given at 2 or 6, but not 10, days after tumour challenge. Successful therapy required: sufficient tumour cells (greater than or equal to 5 X 10(4)); an optimal dose of C. parvum (5-120 mug, increasing with the number of tumour cells); an intact T cell system; the same tumour cells for challenge and treatment. The specificity was confirmed in a protection system in which treatment was given 7 days before tumour challenge. No protective immunity could be achieved with mixtures of C. parvum and foetal cells. Thus in this system C. parvum potentiates protective immunity only to the tumour unique TSTA.


*Br. J. Cancer* (1975) **32**, 551

### ACTIVE SPECIFIC IMMUNOTHERAPY OF MOUSE METHYLCHOLANTHRENE INDUCED TUMOURS WITH *CORYNEBACTERIUM PARVUM* AND IRRADIATED TUMOUR CELLS

R. BOMFORD

*From the Department of Experimental Immunobiology, Wellcome Research Laboratories, Beckenham, Kent BR3 3BS*

Received 2 June 1975. Accepted 7 July 1975

**Summary.—The relative efficiency of active nonspecific or specific immunotherapy of developing methylcholanthrene induced fibrosarcomata with *C. parvum* was compared. For nonspecific immunotherapy, mice were challenged with tumour cells s.c. or i.v., and 2 days later injected i.v. with dilutions of *C. parvum.* The only significant effect was a retardation of s.c. tumour growth by the highest concentration of *C. parvum* (350 μg). However, active specific immunotherapy, using mixtures of *C. parvum* and irradiated or living tumour cells in the footpads, suppressed tumour growth when given at 2 or 6, but not 10, days after tumour challenge. Successful therapy required: sufficient tumour cells ($\geqslant 5\times10^4$); an optimal dose of *C. parvum* (5–120 μg, increasing with the number of tumour cells); an intact T cell system; the same tumour cells for challenge and treatment. The specificity was confirmed in a protection system in which treatment was given 7 days before tumour challenge. No protective immunity could be achieved with mixtures of *C. parvum* and foetal cells. Thus in this system *C. parvum* potentiates protective immunity only to the tumour unique TSTA.**

ACTIVE specific immunotherapy of rodent tumours with irradiated tumour cells can be accomplished when the tumours are small (Haddow and Alexander, 1964), and in some cases therapy is improved if *Bacillus Calmette–Guerin* is given as well (Mathé, Pouillart and Lapeyraque, 1969; Parr, 1972). *Corynebacterium parvum* is an adjuvant in non-tumour systems (reviewed by Howard, Scott and Christie, 1973) and there is evidence that it can, when used in combination with irradiated tumour cells, potentiate the immune response to tumour-specific transplantation antigens (TSTA) (Proctor, Rudenstam and Alexander, 1973; Scott, 1974*a*). This paper describes the use of *C. parvum* and irradiated tumour cells for the immunotherapy of methylcholanthrene induced fibrosarcomata (MC fibrosarcomata) of CBA mice. Some factors controlling the success of therapy are analysed.

### MATERIALS AND METHODS

*Mice.*—Male CBAT6T6 mice aged 8–12 weeks, bred in this department, were used.

C. parvum—A killed saline suspension (Wellcome strain CN6134, batch PX374), prepared by the method of Adlam and Scott (1973), was provided by Wellcome Reagents Ltd, Beckenham, Kent.

*Tumours.*—Tumours appeared in male CBAT6T6 mice 4–5 months after the intramuscular injection in the thigh of 0·1 ml of methylcholanthrene (5 mg/ml in olive oil, kindly provided by Dr J. A. Wright of the Imperial Cancer Research Fund, London), and were maintained by transplantation in male CBAT6T6 mice and in tissue culture. Three independently induced tumours, M2, M3 and M4, were used in these experiments.

*Tumour cell suspensions.*—Cells were obtained directly from tumours by mincing small fragments of non-necrotic tumour tissue in PBS, pushing the fragments through a sieve, allowing the debris to settle and washing the cells in PBS. Viability was 20–30% by trypan blue exclusion.

Tissue culture cells were kept in Dulbecco's modification (containing 4 times the usual amount of amino acids) of Eagle's medium with 10% heat inactivated foetal calf serum (Wellcome Reagents Ltd). They were brought into suspension with 0·1% trypsin in 0·05% versene.

*Foetal calf suspensions.*—10–14 day old foetuses were minced, the fragments stirred in 0·1% trypsin in PBS for 5–10 min at 37°C, the fluid decanted, calf serum added to 10% and spun at 1000 *g* for 5 min. The cell pellet was resuspended in PBS–10% FCS. Viability was >90%.

*Irradiation of cells.*—Cells were exposed to 10,000 rad from a $^{60}$Co source.

*Tumour challenge and therapy.*—0·1 ml (for dorsal s.c. challenge) or 0·2 ml (for i.v. challenge) of tumour derived cells in PBS or tissue culture cells in medium were injected. For therapy, 0·05 ml of dilutions of *C. parvum* and/or tumour cells in saline were injected into both footpads.

Mice challenged s.c. were palpated weekly for tumours until control tumours started to ulcerate and were then killed and the tumours weighed. Mice challenged i.v. were scored for survival up to 80 days, the remaining mice then being killed. Lungs were examined for tumours by the method of Wexler (1966).

*T cell depleted mice.*—Mice were thymectomized, irradiated (850 rad) and reconstituted with $2\times10^6$ bone marrow cells following the method of Davies *et al.* (1966).

*Statistics.*—Statistical manipulation of the data was carried out by Mr D. Field, Wellcome Research Laboratories. The $\log_{10}$ geometric means of tumour weights were compared using a pooled variance estimate from all groups. The arithmetic means of survival times were compared by Student's *t* test. Significance was $P<0·05$.

### RESULTS

### *Effect of* C. parvum *injected alone i.v. against tumour challenge*

Mice were injected i.v. with $2\times10^5$, or s.c. with $10^4$ tissue culture M4 cells, and 2 days later injected i.v. with dilutions of *C. parvum.* Table I shows that the only significant effect was a retardation of s.c. tumour growth by the highest dose of *C. parvum* (350 μg).

### *Therapy using* C. parvum *irradiated tumour cell mixtures: effect of varying doses of* C. parvum *and irradiated cells*

Mice were injected s.c. with $10^4$ tissue cultured M4 cells and treated 2 days later with graded doses of irradiated M4 cells and *C. parvum.* Table II shows that dilutions of *C. parvum* or of irradiated tumour cells given alone did not suppress or significantly retard tumour growth. Mixtures containing the smallest number ($5\times10^3$) of M4 cells were ineffective but with $5\times10^4$ M4 cells therapy was successful at the lower doses of *C. parvum* (1·4 and 5·5 μg) but not at the higher doses (22 and 88 μg), although with 22 μg of *C. parvum* there was a significant reduction of tumour weight (the only one in this experiment). When the number of M4 cells was raised to $5\times10^5$, complete therapy was achieved with 22 μg of *C. parvum,* and partial therapy (only 1/5 mice with tumours) at the other dilutions. The mice which remained tumour-free at 6 weeks had not developed palpable tumours earlier.

Since in this experiment tissue culture M4 cells were used both for tumour challenge and therapy, the possibility that tumour suppression could be due to immunity to tissue culture constituents was excluded by repeating part of the experiment, using $10^6$ irradiated tumour derived M4 cells for therapy. Five/5 mice developed tumours in the untreated controls, and in the groups given M4 cells or *C. parvum* dilutions alone. Therapy with mixtures, as before, was successful only with lower doses of *C. parvum*: 350 μg, 5/5; 88 μg, 4/5; 22 μg, 1/5; 5·5 μg, 0/5.

### *Efficacy of delayed therapy*

Mice were injected s.c. with $10^4$ tissue culture M4 cells 6 or 10 days before treatment with $5\times10^5$ irradiated tissue culture M4 cells, 22 μg *C. parvum,* or mixtures. Table III shows that therapy was successful

TABLE I.—*The Effect of i.v. Treatment with* C. parvum 2 *Days after s.c. or i.v. Challenge with M*4 *Cells*

| Amount *C. parvum* (μg) | s.c. challenge ($10^4$ cells): Proportions mice with tumours at 6 weeks | s.c. challenge: Tumour weights (mg, $\log_{10}$ geometric mean ± s.e.) | i.v. challenge ($2 \times 10^5$ cells): Proportions mice dead at 80 days | i.v. challenge: Survival time (days, arithmetic mean ± s.e.) |
|---|---|---|---|---|
| None | 6/7 | 2·36±0·42 | 6/7 | 49·6±4·9 |
| 350 | 5/5 | 1·36±0·29* | 6/7 | 55·7±3·2 |
| 88 | 5/5 | 1·89±0·36 | 5/7 | 51·1±5·3 |
| 22 | 5/5 | 2·26±0·18 | 7/7 | 55·3±3·7 |
| 5·5 | 5/5 | 2·29±0·11 | 7/7 | 50·1±5·5 |
| 1·4 | 5/5 | 2·49±0·08 | 5/7 | 43·7±4·1 |

* $P<0{\cdot}05$, compared with untreated controls.

TABLE II.—*Footpad Treatment of Mice with* C. parvum, *Irradiated M*4 *Cells or Mixtures,* 2 *Days after s. c. Challenge with* $10^4$ *M*4 *Cells. The Effect of varying the Dose of* C. parvum *and Number of Tumour Cells*

Proportion of mice with tumours, and tumour weights (mg, $\log_{10}$ geometric mean ± s.e.)

| No. of irradiated M4 cells | Amount of *C. parvum* (μg): None | 1·4 | 5·5 | 22 | 88 |
|---|---|---|---|---|---|
| None | 4/5<br>2·21±0·15 | 5/5<br>1·80±0·11 | 5/5<br>1·39±0·30 | 4/5<br>1·74±0·20 | 5/5<br>1·88±0·11 |
| $5\times10^3$ | 5/5<br>2·22±0·19 | 5/5<br>2·02±0·21 | 5/5<br>1·85±0·11 | 4/5<br>2·23±0·30 | 4/5<br>2·02±0·44 |
| $5\times10^4$ | 5/5<br>1·54±0·35 | 0/5 | 0/5 | 5/5<br>0·97±0·18* | 5/5<br>2·02±0·17 |
| $5\times10^5$ | 5/5<br>2·34±0·21 | 1/5<br>1·76 | 1/5<br>2·22 | 0/5 | 1/5<br>2·23 |

* $P<0{\cdot}05$, compared with untreated controls.

TABLE III.—*Footpad Treatment of Mice with* C. parvum, *Irradiated M*4 *cells or Mixtures* 6 *or* 10 *Days after s.c. Challenge with* $10^4$ *M*4 *Cells*

| | Day 6 treatment: Expt 1 | Day 6 treatment: Expt 2 | Day 10 treatment: Expt 1 |
|---|---|---|---|
| Untreated controls | Not done | 5/5 (2·03±0·77) | Not done |
| *C. parvum* (22 μg) | 4/5 (1·69±0·16) | 5/5 (2·08±0·85 | 4/5 (2·45±0·79) |
| M4 cells ($5\times10^5$) | 5/5 (2·29±0·17) | 5/5 (1·72±0·28) | 5/5 (2·28±0·11) |
| *C. parvum* plus M4 cells | 0/5 | 1/5 (1·53) | 4/5 (1·83±0·43) |

at 6 days after tumour challenge, when no convincingly palpable tumours could be found. In the first experiment one tumour was palpable at 14 days after s.c. challenge, but not at Day 21; in the second experiment all 5 tumours were palpable at Day 14, and 4 out of 5 had disappeared at Day 21.

Therapy 10 days after s.c. challenge, when the tumours were already palpable, was ineffective.

*The effect of T cell depletion*

Mice depleted of T cells by thymectomy, irradiation and reconstitution with bone marrow cells (TX-IRR-BM) and irradiated reconstituted controls (IRR-BM) were challenged s.c. with $10^4$ M4 cells and treated 2 days later with $5\times10^5$ irradiated tissue culture M4 cells, 5·5 μg *C. parvum* or a mixture (Table IV). Therapy was impaired in T cell depleted mice.

TABLE IV.—*Footpad Treatment with* C. parvum *Irradiated Tumour Cells or Mixtures, 2 days after s.c. Challenge with* $10^4$ *M4 Cells in T Cell Depleted (TX-IRR-BM) or Control (IRR-BM) Mice*

| | Proportion of mice with tumours and tumour weights (mg, $\log_{10}$ geometric mean ± s.e.) | |
|---|---|---|
| | IRR-BM | TX-IRR-BM |
| Untreated controls | 4/4 (1·71±0·12) | 4/4 (1·75±0·37) |
| *C. parvum* (5·5 μg) | 4/4 (2·08±0·05) | 4/4 (2·16±0·09) |
| M4 cells (5×$10^5$) | 4/4 (1·95±0·15) | 4/4 (1·92±0·10) |
| *C. parvum* plus M4 cells | 1/4 (0·70) | 4/4 (1·74±0·36) |

TABLE V.—*The Specificity of Footpad Therapy with* C. parvum *and Irradiated Tumour Cells*

| | Proportion of mice with tumours and tumour weight (mg, $\log_{10}$ geometric mean±s.e.) | |
|---|---|---|
| | M3 challenge | M4 challenge |
| Untreated controls | 5/5 (2·10±0·21) | 4/5 (1·56±0·09) |
| *C. parvum* (22 μg) | 5/5 (1·87±0·31) | 2/5 (1·23±0·53) |
| M3 cells (5×$10^5$) | 4/5 (2·29±0·49) | 3/5 (1·82±0·08) |
| M4 cells (5×$10^5$) | 5/5 (2·40±0·61) | 3/5 (2·09±0·37) |
| *C. parvum* plus M3 cells | 0/5 | 3/5 (1·80±0·12) |
| *C. parvum* plus M4 cells | 4/5 (2·17±0·31) | 0/5 |

Challenge with independently induced M3 or M4 tumour cells and therapy 2 days later with homologous or heterologous cells.

TABLE VI.—*Specificity of the Protective Effect of Footpad Treatment with* C. parvum *and Irradiated Tumour Cells. Treatment given 7 Days before s.c. Challenge with* $10^4$ *M4 Cells*

| Amount of *C. parvum* (μg) | Proportion of mice with tumours and tumour weights (mg, $\log_{10}$ geometric mean ± s.e.) — Cells in treatment mixture: None | M3 | M5 |
|---|---|---|---|
| None | 5/5<br>2·06±0·11 | 5/5<br>2·29±0·06 | 3/5<br>1·45±0·22 |
| 22 | 5/5<br>1·94±0·31 | 5/5<br>1·80±0·29 | 0/5 |
| 5·5 | 5/5<br>2·07±0·15 | 5/5<br>2·16±0·13 | Not done |
| 1·4 | 5/5<br>1·52±0·22 | 5/5<br>2·15±0·12 | Not done |

*Specificity of therapy*

Mice were injected s.c. with 1·5 × $10^4$ M4 cells or 2 × $10^4$ M3 cells and 2 days later received therapy with 5 × $10^5$ irradiated tissue culture M3 or M4 cells, 22 μg *C. parvum* or mixtures (Table V). Homologous cells were required in therapy mixtures.

The specificity of therapy was confirmed using i.v. challenge with tumour cells. Mice were injected i.v. with 2 × $10^5$ M4 or M2 cells and 2 days later injected in the footpads with 120 μg of *C. parvum* and $10^6$ living M2 or M4 cells. All the mice treated with *C. parvum* and homologous tumour cells were without lung nodules when killed 80 days after i.v. challenge. Treatment with mixtures containing heterologous cells did not prolong survival (M4 cell challenge, mean survival time, days ± s.e., untreated controls 49·0 ± 3·6, *C. parvum*-M2 cell treatment $54 \cdot 9 \pm 4 \cdot 8$; M2 cell challenge, untreated controls $35 \cdot 2 \pm 1 \cdot 2$, *C. parvum*-M4 cell treatment $38 \cdot 6 \pm 1 \cdot 5$).

To increase the chances of detecting a low level of cross reactive anti-tumour resistance, further specificity experiments were performed using a protection model in which treatment preceded tumour challenge.

Mice were injected in the footpads with $10^6$ irradiated tissue culture M3 or M4 cells, with dilutions of *C. parvum* or with mixtures, and were challenged s.c. 7 days later with $4 \times 10^4$ viable tumour derived M3 or M4 cells. Table VI shows the results of the M4 challenge experiment. Prior treatment with M4 cells alone conferred partial resistance, which became complete in conjunction with 22 μg of *C. parvum.* Heterologous M3 cells alone or admixed with 22, 5·5 or 1·4 μg of *C. parvum* did not generate resistance.

The reciprocal experiment using M3 cells for s.c. challenge gave analogous results; only M3–*C. parvum* mixtures were protective (results not shown).

*The use of foetal cells*

*C. parvum*–foetal cell mixtures were tested in the protection model. Up to $3 \times 10^6$ lining or irradiated syngeneic (CBA) or allogeneic (BALB/c) 10–14 day foetal cells were injected in the footpads with varying doses of *C. parvum* 7 days before s.c. challenge with M3 or M4 cells. No suppression or retardation of tumour growth was achieved.

### DISCUSSION

The present results show that developing MC fibrosarcomata can be eliminated after treatment with mixtures of *C. parvum* and irradiated or living tumour cells. The following factors affected the outcome of treatment.

Sufficient tumour cells are required in the mixtures, the minimum for irradiated cells lying between $5 \times 10^3$ and $5 \times 10^4$. No upper limit was detected, as therapy was successful with the largest number tested, $10^6$ living cells. Sufficient but not excessive *C. parvum* is required, effective doses lying between 1·4 and 120 μg, and increasing with the number of tumour cells. These dose requirements are in contrast to those for therapy with i.v. *C. parvum* alone where only the highest dose (350 μg) retarded tumour growth. A similar distinction in dose requirements has previously been described for the P-185 mastocytoma growing on the footpad of C57Bl × DBA/2 mice, where retardation of tumour growth could be best achieved with a high dose (700 μg) of *C. parvum* i.v. (Scott, 1974*b*), whereas regressions after intralesional injections were more frequent with lower (35 or 70 μg) than higher (350 μg) doses (Scott, 1974*a*). It has been suggested that higher doses of *C. parvum* are ineffective for intralesional therapy because the draining nodes become too severely disorganized by histiocytic infiltration to mount an immune response to tumour antigens (Scott, 1974*c*). However, the observation in this paper that the upper limit for the dose of *C. parvum* in mixtures increases with the number of tumour cells suggests an explanation based on the necessity for a balance between *C. parvum* and tumour antigen.

An intact T cell system was required for therapy. This, together with the exquisite specificity of the system, suggests that *C. parvum* is promoting immunity to the non-cross-reacting TSTA. It remains to be determined whether cell mediated or humoral immunity is involved, but cell mediated must be favoured since it has already been shown for the P-185 mastocytoma in C57Bl × DBA/2 mice that lymph node cells, but not serum, from mice pretreated with *C. parvum* and irradiated tumour cells prevents the outgrowth of admixed tumour cells transferred to a sub-lethally irradiated recipient (Scott, 1975).

Although therapy given 6 days after tumour challenge suppressed tumour growth, therapy at 10 days did not even retard tumour growth. This suggests there is a critical tumour size, beyond which therapy must fail, due either to the logarithmic growth of the tumour outstripping the constant destruction of tumour cells by the immune system or to blocking factors.

Therapy or protective immunity required the use of the same tumour cells for treatment and challenge; there was no evidence that *C. parvum*, as used in this study can potentiate immunity (of a type conferring transplantation resistance) to the cross-reacting foetal antigens known to exist on mouse and rat MC-fibrosarcomata (Braun, 1970; Baldwin, Glaves and Vose, 1972; Thomson and Alexander, 1973; LeMevel and Wells, 1973).

The implications of the animal experimental work for the use of *C. parvum* as an immunotherapeutic agent in man have been reviewed by Scott (1974*c*), but some comments specifically related to the present work can be made. If *C. parvum* is used with irradiated tumour cells for active specific immunotherapy, the number of tumour cells used may not be critical, although it would be justifiable to use the maximum feasible so as not to fall below the antigen threshold. Care must be taken to avoid using excessive *C. parvum*. In order to circumvent *C. parvum* overdose it would be preferable to apply repeated treatments to areas drained by different lymph nodes. If no tumour cells are available, active specific immunotherapy may still be attempted by injecting *C. parvum* on its own at a site where it will stimulate a draining lymph node which is likely to be already containing tumour antigen. It has been shown in the P815 mastocytoma system that *C. parvum* and irradiated tumour cells injected at different sites can generate protective immunity, provided that they stimulate the same lymph node (Scott, 1975). In the light of the specificity data presented in this paper, it will be preferable to use irradiated autochthonous tumour cells whenever possible unless there is good evidence for cross-reacting antigens of a type capable of stimulating anti-tumour resistance.

Finally, although it has now been demonstrated in this system and for the P-815 mastocytoma (Scott, 1974*a*, *b*) that i.v. treatment with *C. parvum* produces a relatively weak anti-tumour effect, this method of administering *C. parvum* should not be discounted for clinical use. Firstly, it might enable *C. parvum* to come into contact with, and stimulate the immune response to, systemically distributed tumour cells at sites such as the liver or bone marrow. Secondly, since i.v. *C. parvum* produces an anti-tumour effect which is independent of the immune response to TSTA (Scott, 1974*c*), it might provide some resistance to tumour growth in situations where the possibility of active specific immunotherapy is diminished either by lack of antigenicity of the tumour or by immunological energy of the patient.

The author thanks Mr S. Wishart for excellent technical assistance, and Drs M. Scott and J. Ivanyi for helpful criticism of the manuscript.

### REFERENCES

Adlam, C. & Scott, M. T. (1973) Lympho-reticular Stimulatory Properties of *Corynebacterium parvum* and Related Bacteria. *J. med. Microbiol.*, **6**, 261.

Baldwin, R. W., Glaves, D. & Vose, B. M. (1972) Embryonic Antigen Expression in Chemically Induced Rat Hepatomas and Sarcomas. *Int. J. Cancer*, **10**, 233.

Braun, R. J. (1970) Possible Association of Embryonal Antigen(s) with Seral Primary 3-methyl cholanthrene-induced Murine Sarcomas. *Int. J. Cancer*, **6**, 245.

Davies, A. J. S., Leuchars, E., Wallis, V. & Koller, P. C. (1966) The Mitotic Response of Thymus-derived Cells to Antigenic Stimulus. *Transplantation*, **4**, 438.

Haddow, A. & Alexander, P. (1964) An Immunological Method of Increasing the Sensitivity of Primary Sarcomas to Local Irradiation with X-rays. *Lancet*, i, 452.

Howard, J. G. Scott, M. T. & Christie, G. H. (1973) Cellular Mechanisms underlying the Adjuvant Activity of *Corynebacterium parvum*: Interactions of Activated Macrophages with T and B Lymphocytes. In *Immunopotentiation*. Ed. J. W. Wolsterholme & J. Knight. Ciba Foundation Symposium, **18**, 101, Amsterdam: Associated Scientific Publishers.

LeMevel, B. P. & Wells, S. A. (1973) Foetal Antigens Cross-reactive with Tumour-specific transplantation Antigens. *Nature, New Biol.*, **244**, 183.

MATHÉ, G., POUILLART, P. & LAPEYRAQUE, F. (1969) Active Immunotherapy of L1210 Leukaemia Applied after the Graft of Tumour Cells, *Br. J. Cancer*, **23**, 814.

PARR, I. (1972) Response of Syngeneic Immune Lymphomata to Immunotherapy in Relation to the Antigenicity of the Tumour. *Br. J. Cancer*, **26**, 174.

PROCTOR, J., RUDENSTAM, C. M. & ALEXANDER, P. (1973) Increased Incidence of Lung Metastases following Treatment of Rats bearing Hepatomas with Irradiated Tumour Cells and the Beneficial Effect of *Corynebacterium parvum* on this System. *Biomedicine*, **19**, 248.

SCOTT, M. T. (1974*a*) *Corynebacterium parvum* as a Therapeutic Antitumor Agent in Mice. II. Local Injection. *J. natn. Cancer Inst.*, **53**, 861.

SCOTT, M. T. (1974*b*) *Corynebacterium parvum* as a Therapeutic Antitumor Agent in Mice. I. Systemic Effects from Intravenous Injection. *J. natn. Cancer Inst.*, **53**, 855.

SCOTT, M.T. (1974*c*) *Corynebacterium parvum* as an Immunotherapeutic Anticancer Agent. *Semin. Oncol.*, **1**, 367.

SCOTT, M. T. (1975) Potentiation of the Tumor-specific Immune Response by *Corynebacterium parvum*. *J. natn. Cancer Inst.*, **55**, 65.

THOMSON, D. M. P. & ALEXANDER, P. (1973) A Cross-reacting Embryonic Antigen in the Membrane of Rat Sarcoma Cells which is Immunogenic in the Syngenic Host. *Br. J. Cancer*, **27**, 35.

WEXLER, H. (1966) Accurate Identification of Experimental Pulmonary Metastases. *J. natn. Cancer Inst.*, **36**, 641.